# Initiation of aripiprazole once-monthly via a two‑injection start in adult patients diagnosed with schizophrenia: real-world experiences of healthcare professionals from five European countries

**DOI:** 10.1186/s12991-026-00684-z

**Published:** 2026-05-31

**Authors:** Clodagh Beckham, Murat Yildirim, Andrea Fagiolini, Karolina Leopold, William J. Cottam, Joe Hickey, Olivia Rogerson, Sofia Pappa

**Affiliations:** 1 Otsuka Pharmaceutical Europe Ltd., Berkshire, UK; 2 H. Lundbeck A/S, Ottiliavej 9, Valby, 2500 Denmark; 3 Università di Siena, Siena, Italy; 4 Vivantes Hospital am Urban and Vivantes Hospital im Friedrichshain, Charite, Universitätsmedizin, Berlin Germany; 5 Carl Gustav Carus University Hospital, Technische Universität Dresden, Dresden, Germany; 6 Real‑World Evidence, OPEN Health, London, UK; 7 Department of Brain Sciences, Division of Psychiatry, Imperial College London, London, UK

**Keywords:** (7/10): Clinical practice, Long-acting injectable, Real-world evidence, Survey, Aripiprazole once-monthly, Two-injection start, 1-day initiation regimen

## Abstract

**Background:**

The aripiprazole once-monthly 400 mg two-injection start (AOM 400-TIS) enables treatment initiation at a single clinic visit, without the need for 14 days of concurrent oral aripiprazole supplementation. Previously, results from a survey outlined the views and experiences of healthcare professionals (HCPs) with the AOM 400-TIS in a real-world setting in Europe. The current work extends these findings to include a broader pool of participants from more European countries.

**Methods:**

This was a non-interventional, cross-sectional survey of HCPs from Germany, Italy, the United Kingdom, Denmark, and Sweden. Participants were licensed nurses/physicians with experience prescribing and/or administering the AOM 400-TIS to patients diagnosed with schizophrenia. Two waves of survey data were pooled and analysed using descriptive methods.

**Results:**

Data from 229 HCPs were evaluated. Poor treatment adherence (88.6%), relapse (51.1%), and patient preference (47.6%) were common reasons for using the AOM 400-TIS, while patients not wanting two injections at the same time (52.4%) and concerns about safety (34.1%) and tolerability (33.6%) were common barriers. Among HCPs, 86.0% agreed/strongly agreed they were satisfied with outcomes of patients treated with the AOM 400-TIS, with most agreeing/strongly agreeing that patients appeared satisfied in general (73.4%) and with sustained quality of life and functioning (66.4%).

**Conclusions:**

This survey provides a pan-European account of HCPs’ views and experiences with the AOM 400-TIS in adults diagnosed with schizophrenia. Initiatives to overcome barriers to AOM 400-TIS use include providing clearer evidence and education on its efficacy and safety and consideration of how the regimen is presented in patient−provider discussions.

**Supplementary Information:**

The online version contains supplementary material available at 10.1186/s12991-026-00684-z.

## Introduction

Long-acting injectable (LAI) antipsychotics are considered a valuable tool for the treatment of schizophrenia [1]. LAI antipsychotics are administered at intervals ranging from weeks to months [2], and were developed to improve medication adherence by eliminating the need for daily dosing with oral antipsychotics [3]. Studies have demonstrated greater adherence to/persistence with LAI versus oral antipsychotic formulations [4, 5], along with improved outcomes, including a lower rate of relapse [6], reduced healthcare resource utilisation (i.e., emergency room visits, all-cause and psychiatric hospitalisations) [4–6], lower healthcare costs [4], and a reduced risk of all-cause mortality [7]. By supporting treatment adherence, easing disease and treatment burden, and reducing functional decline, there is consensus that LAIs can also facilitate greater functional recovery [8].

Aripiprazole monohydrate LAIs are available as a once-monthly injection (aripiprazole once-monthly 400 mg [AOM 400]) and, more recently, as an injection administered every other month (aripiprazole 2-month ready-to-use 960 mg [Ari 2MRTU 960]) [9–11]. AOM 400 is approved in Europe for the maintenance treatment of adult patients diagnosed with schizophrenia who are stabilised with oral aripiprazole [12, 13]. AOM 400 can be initiated in one of two ways: as a single injection of AOM 400 plus 14 consecutive days of supplemental oral aripiprazole; or as two injections of AOM 400 at the same time (but at different anatomical sites) plus a single dose of oral aripiprazole 20 mg, all administered on the same day during a single clinic visit [12, 13]. In a clinical setting, initiation of AOM 400 with two injections is generally referred to as the two-injection start (TIS) or the 1-day initiation regimen [14].

The AOM 400-TIS represents a simplified and straightforward start to treatment with AOM 400 [15, 16] that offers several potential benefits to patients, healthcare professionals (HCPs), and caregivers [15]. Specifically, since the regimen results in therapeutic drug levels on the first day of treatment without the need for 14 days of oral aripiprazole supplementation, it reduces the risk of relapse arising from suboptimal adherence to oral aripiprazole [15]. Related to this, it reduces the burden of daily tablet taking for patients in the initial treatment period, and any associated burden on the part of HCPs, families, and/or other caregivers to supervise oral medication intake [15]. In hospitalised patients, this may allow for an earlier discharge [15]. Lastly, and more broadly, the availability of the AOM 400-TIS provides patients and HCPs with greater treatment choice [15], which aligns with a recent qualitative interview study, where researchers emphasised the importance of shared decision-making and the need for prescribers to engage patients in conversation to explore any preconceptions they might have about LAIs [17].

The AOM 400-TIS has been available in Europe since October 2020. However, evidence on the real-world implementation of the AOM 400-TIS was limited [18]. In response, researchers conducted an initial survey of 94 HCPs from Germany, Italy, and the United Kingdom (UK) to gather information on their views of, and experiences with, the AOM 400-TIS in adult patients diagnosed with schizophrenia (“Wave 1”) [18]. Per participant feedback, HCPs most often prescribed the AOM 400-TIS to improve adherence and reduce the risk of relapse, while the most common barriers to its use were patient reluctance and concerns regarding safety/tolerability [18]. To extend the geographical reach of the survey, increase its representativeness, and gather additional insight, the survey was extended (“Wave 2”) to include feedback from HCPs from Denmark and Sweden, and additional HCPs from Italy, a country in which, in the authors’ view, uptake of the AOM 400-TIS has been relatively high. The aim of the current manuscript is to present the previously unpublished data from Wave 2 of the survey, pooled with findings from Wave 1, providing a pan-European account of HCPs’ views and experiences with the AOM 400-TIS in a real-world setting. Overall, it was hypothesized that Wave 2 of the survey would confirm findings from Wave 1.

## Materials and methods

### Study design and objectives

This was a non-interventional, cross-sectional survey of HCPs that was undertaken in two parts: Wave 1 (1 February–21 March 2024) included participants from Germany, Italy, and the UK, while Wave 2 (18 September–27 November 2024) included participants from Denmark, Sweden, and Italy (separate to those who participated in Wave 1). The primary objective of the survey was to explore HCPs’ experiences and satisfaction with the AOM 400-TIS [18]. Secondary objectives were to: characterise the HCPs who use the AOM 400-TIS; explore patient- and clinical-related factors influencing their decision to initiate treatment this way; assess their views on the efficacy, safety, and tolerability of the AOM 400-TIS; and capture broader perspectives on use of the AOM 400-TIS in clinical practice [18].

### Sample population and survey format

A full and thorough description of the sample population and survey format has been reported previously [18]. In brief, eligible participants were licensed nurses or physicians involved in the treatment and management of schizophrenia who were experienced in prescribing and/or administering the AOM 400-TIS to patients diagnosed with schizophrenia [18]. Individuals who were employed by a pharmaceutical company were unable to participate [18].

Invited and interested individuals who fulfilled the study eligibility criteria (including provision of electronic informed consent) completed a secure, web-based survey in their local language [18]. The survey, designed to take 15–20 min to complete, asked HCPs to reflect on their past experience with the AOM 400-TIS in adult patients diagnosed with schizophrenia, administered according to the label in the European Union or the UK, as appropriate [18]. The majority of questions had set answer choices, with participants selecting one or more responses, as instructed [18].

The survey was developed with input from a steering committee of clinical experts to ensure that the questions reflected real-world European practice and that the sample represented relevant settings and specialties involved in prescribing or administering AOM 400-TIS [18]. Data were collected in a confidential and pseudonymised manner [18]. A full version of the survey, encompassing questions presented to participants in Waves 1 and 2 of the survey, is shown in Supplementary Table 1. Survey participants were compensated for their time with a modest payment in their local currency [18].

Participant recruitment in Wave 1 involved random selection of individuals who were enrolled in online panels/databases and who had expressed an interest in participating in research studies [18]. Participants were recruited in the same way in Wave 2 (i.e., via online panels), with extra Italian participants also recruited on a volunteer basis via the local network of the Italian member of the steering committee.

### Statistical analysis

Owing to the descriptive nature of the study, a formal power calculation was deemed unnecessary; instead, sample sizes of up to 90 and 140 HCPs in Waves 1 and 2, respectively, were selected. The plan was to recruit up to 30 HCPs per country from Germany (Wave 1), the UK (Wave 1), Denmark (Wave 2), and Sweden (Wave 2), and up to 110 HCPs from Italy (Wave 1, *n* = 30; Wave 2, *n* = 80 [*n* = 50 recruited via online panels and *n* = 30 recruited via the Italian steering committee member]). For online panel-based recruitment, the target distribution was ~ 20 physicians and ~ 10 nurses per country from Germany, the UK, Denmark, and Sweden, and ~ 55 physicians (Wave 1, *n* = 20; Wave 2, *n* = 35) and ~ 25 nurses (Wave 1, *n* = 10; Wave 2, *n* = 15) from Italy. There was no planned distribution of physicians versus nurses in the subset of Italian participants who were recruited on a volunteer basis.

Responses to each survey item were summarised descriptively on an item-by-item basis according to the responses received using Microsoft^®^ Excel^®^ 365. Only the data provided by participants were analysed, with no imputation of missing data [18]. Endpoints of interest (Table 1) were not formally prespecified, and no statistical testing was performed.

**Table 1:** Summary of endpoints of interest

Endpoint	Metric
Most common reason(s) selected by HCPs for initiating maintenance treatment with the AOM 400-TIS	Proportion of HCPs (%)
Main objective(s) selected by HCPs when choosing to prescribe the AOM 400-TIS initiation regimen	
Reasons and/or barriers selected by HCPs for not using the AOM 400-TIS initiation regimen	
Important patient and clinical factors that influence HCPs’ decision to prescribe the AOM 400-TIS initiation regimen	

AOM 400-TIS: aripiprazole once-monthly 400 mg two-injection start; HCP: healthcare professional

## Results

**Fig. 1:**
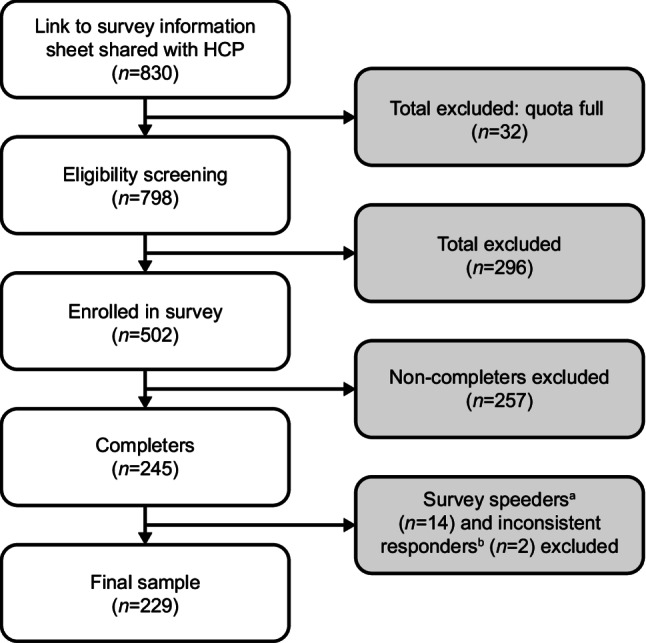
Flow of participants through Waves 1 and 2 of the study. ^a^Completed the survey in ≤ 4 min [18]. ^b^Excluded at analysis stage due to inconsistent reporting, which was deemed to compromise the validity of the responses. HCP: healthcare professional

Across Waves 1 and 2, 798 HCPs completed the screening questions, with 502 HCPs subsequently enrolled into the survey. Of these, 245 HCPs completed the survey, although responses from 16 HCPs were subsequently excluded (‘speeders’ [i.e., completed the survey in ≤ 4 minutes], *n* = 14; inconsistent responders, *n* = 2). The final analysis set included 229 HCPs (Fig. 1), comprising 28 participants from Denmark, 30 from Sweden, 31 from Germany, 32 from the UK, and 108 from Italy.

### Participant characteristics

The characteristics of the survey participants in the overall cohort are shown in Table 2. Participants were mostly psychiatrists (66.4%) or psychiatric nurses (27.1%), and their primary work environment was most often a specialist mental health clinic/centre and/or a hospital inpatient setting. The mean number of years that participants had worked professionally in clinical practice was 19.2 years (range: 1–69 years of professional experience). Among the patients currently under the care of participating HCPs, a mean (standard deviation [SD]) of 35.6% (24.6%) had a diagnosis of schizophrenia; of these, a mean (SD) of 45.0% (24.0%) were treated with LAI antipsychotics. A breakdown of participant characteristics according to country is shown in Supplementary Table 2.

**Table 2:** Characteristics of HCP survey participants

Characteristics	*N* = 229
Country of work, *n* (%)	
Denmark	28 (12.2)
Germany	31 (13.5)
Italy	108 (47.2)
Sweden	30 (13.1)
United Kingdom	32 (14.0)
Primary job role/specialty, *n* (%)	
Physician	
General practitioner/primary care practitioner	2 (< 1)
Psychiatrist	152 (66.4)
Neurologist	0 (0)
Other^a^	3 (1.3)
Nurse	
Psychiatric	62 (27.1)
Community	8 (3.5)
Other^b^	2 (< 1.0)
Primary work setting, *n* (%)^c^	
Specialist mental health clinic/centre	152 (66.4)
Hospital inpatient	94 (41.0)
Hospital outpatient	69 (30.1)
General practitioner	19 (8.3)
Other^d^	9 (3.9)
Time working professionally in clinical practice, median (IQR) [range], years	19.0 (11.0–25) [1.0–69]
Proportion of patients in caseload diagnosed with schizophrenia, median (IQR) [range], %	30.0 (18.0–50) [3.0–100]
Proportion of patients in caseload diagnosed with schizophrenia and treated with LAIs, median (IQR) [range], %	50.0 (25.0–65) [0–100]
Main responsibility regarding the AOM 400-TIS, n (%)	
Prescribing	104 (45.4)
Administration	61 (26.6)
Both	64 (27.9)

^a^Other responses included: medical resident (*n* = 1); physician under training in psychiatry (*n* = 1); and psychiatry resident (*n* = 1)

^b^Other responses included: ward physician (*n* = 1); and hospital (*n* = 1)

^c^Participants could select all response options that applied

^d^Other responses included: “provide specialist second opinions for treatment” (*n* = 1); “mental health centre” (*n* = 1); “psychiatric practice” (*n* = 1); “specialist practice” (*n* = 1); “private clinic” (*n* = 1); “outpatient clinic, rehabilitation facilities” (*n* = 1); “psychiatric diagnosis and treatment unit (Servizio Psichiatrico di Diagnosi e Cura)”^e^ (*n* = 1); “psychosocial centre” (*n* = 1); and “university psychiatry department” (*n* = 1)

^e^Servizio Psichiatrico di Diagnosi e Cura is an Italian hospital unit where voluntary and mandatory inpatient psychiatric treatment is provided; it is based within hospital facilities and it is part of the mental health department

AOM 400-TIS: aripiprazole once-monthly 400 mg two-injection start; HCP: healthcare professional; IQR: interquartile range; LAI: long-acting injection

### Current use of the AOM 400-TIS in a maintenance treatment setting

Table 3 outlines factors related to HCP use of the AOM 400-TIS. Over one-half of participants (55.5%) stated that they had first started prescribing and/or administering the AOM 400-TIS more than 24 months ago. Per HCP feedback, the AOM 400-TIS was most often used in patients with moderate and/or severe symptoms. It was most commonly prescribed to adults aged 18–35 and 36–64 years, with relatively limited use in patients aged 65 years or older. A per country breakdown of factors related to HCP use of the AOM 400-TIS is shown in Supplementary Table S3. There was a trend for greater familiarity with the AOM 400-TIS in Nordic countries versus other regions of Europe, along with an overall shorter period of oral aripiprazole stabilisation prior to implementing the AOM 400-TIS. Compared with HCPs from other countries, those from Germany were less likely to have prescribed the AOM 400 TIS for > 24 months, were less likely to select an oral aripiprazole stabilisation period of < 14 days prior to implementing the AOM 400-TIS, and were less likely to prescribe the AOM 400-TIS in an inpatient setting.

**Table 3:** HCP use of the AOM 400-TIS initiation regimen in a maintenance treatment setting

	*N* = 229
Approximate time since HCP started to prescribe/administer the AOM 400-TIS, *n* (%)	
< 6 months ago	27 (11.8)
6–12 months ago	35 (15.3)
13–24 months ago	40 (17.5)
> 24 months ago	127 (55.5)
Average time spent by patient on oral aripiprazole prior to initiating AOM via the AOM 400-TIS, n (%)	
< 14 days	87 (38.0)
14–28 days	93 (40.6)
> 28 days	49 (21.4)
Average severity of symptoms of typical patients whose treatment with AOM was initiated via the AOM 400-TIS, n (%)^a^	
Mild	43 (18.8)
Moderate	150 (65.5)
Severe	117 (51.1)
Age of typical (adult) patients whose treatment with AOM was initiated via the AOM 400-TIS, n (%)^a^	
18–35 years	162 (70.7)
36–64 years	148 (64.6)
≥ 65 years	15 (6.6)
Treatment setting in which the AOM 400-TIS is typically prescribed, n (%)^a^	
Inpatient	150 (65.5)
Outpatient	144 (62.9)
Other^b, c^	3 (1.3)

^a^Participants could select all response options that applied

^b^This response option was only included in Wave 2 of the survey

^c^Other responses included: “both” (*n* = 2); and “a constant carer is available” (*n* = 1)

AOM: aripiprazole once-monthly; AOM 400-TIS: aripiprazole once-monthly 400 mg two-injection start; HCP: healthcare professional

**Fig. 2:**
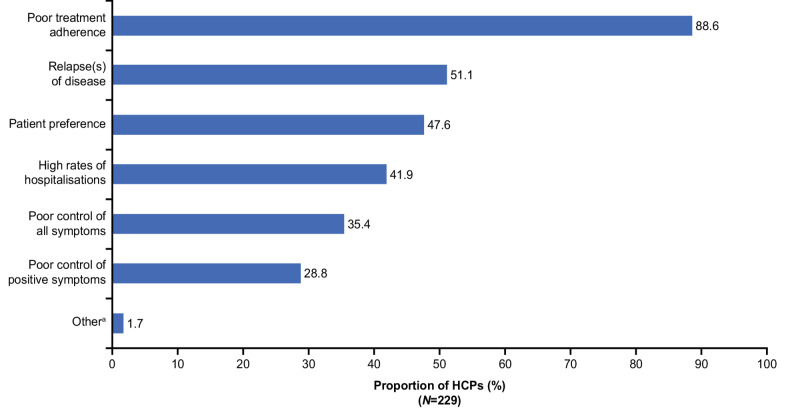
Most common reason(s) selected by HCPs for initiating maintenance treatment with the AOM 400-TIS. Data are ranked from the most to the least selected response options. Participants could select all response options that applied. ^a^Other responses included: “side effect profile” (*n* = 1); “simplification of the dosage scheme” (*n* = 1); “improved medication efficacy and tolerability” (*n* = 1); and “less degree of disease symptoms when taking pills daily” (*n* = 1). AOM 400-TIS: aripiprazole once-monthly 400 mg two-injection start; HCP: healthcare professional

Figure 2 displays the most frequently reported reasons that HCPs chose to initiate treatment with the AOM 400-TIS. Of the available responses, “Poor treatment adherence” was selected most frequently (88.6%), followed by “relapse(s) of disease” (51.1%), and “patient preference (i.e., patient not willing to take oral medication)” (47.6%). A per country breakdown of reasons for initiating the AOM 400-TIS is shown in Supplementary Fig. 1.

**Fig. 3:**
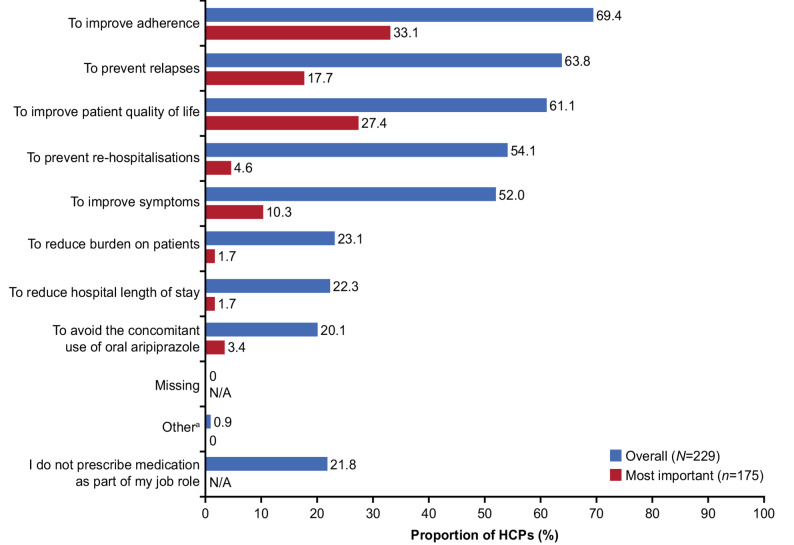
Main objective(s) selected by HCPs when choosing to prescribe the AOM 400-TIS initiation regimen. Participants were asked to select all response options that applied (overall category), and to select one response option that they considered the most important (most important category). The denominator was *N* = 229 for the overall category (all participants) and *n* = 175 for the most important category (all participants [*N* = 229] minus those who did not prescribe as part of their job role [*n* = 50] and those with missing responses [*n* = 4]). Data are ranked from the most to least selected response options in the overall category. In the overall category, participants could select all response options that applied. ^a^Other responses included: “improving tolerability” (*n* = 1) and ‘stabilise serum values and hopes of less adverse reaction fluctuations” (*n* = 1). AOM 400-TIS: aripiprazole once-monthly 400 mg two-injection start; HCP: healthcare professional; N/A: not applicable

Figure 3 displays the primary objectives cited by HCPs when choosing to prescribe the AOM 400-TIS, with “to improve adherence” (69.4%), “to prevent relapses” (63.8%), and “to improve patient quality of life” (QoL, 61.1%) being most often selected. The same three factors were selected when HCPs were prompted to identify the single most important aspect in their choice to prescribe the AOM 400-TIS, with the greatest priority given to improving adherence. A per country breakdown of HCP objectives for choosing the AOM 400-TIS is shown in Supplementary Fig. 2.

**Fig. 4:**
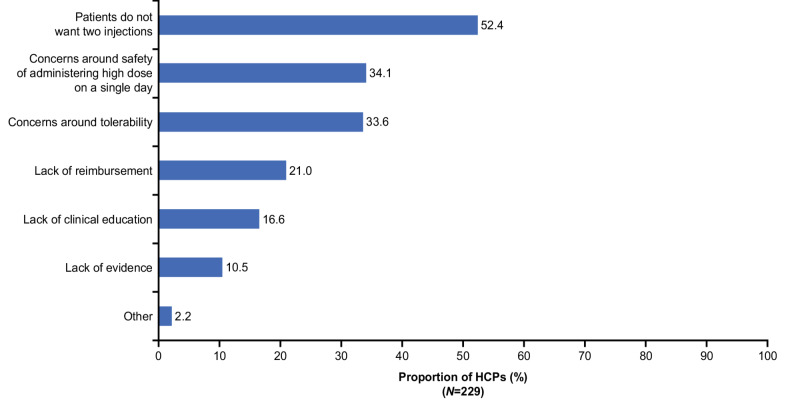
Reasons and/or barriers selected by HCPs for not using the AOM 400-TIS initiation regimen. Data are ranked from the most to least selected response options. Participants could select all response options that applied. ^a^Other responses included: “based on how the question is phrased, I have no obstacles but other colleagues may not prescribe due to lack of specific training” (*n* = 1); “none” (*n* = 1); “I do not prescribe” (*n* = 1); “Nothing” (*n* = 1); “I administer by prescription” (*n* = 1). AOM 400-TIS: aripiprazole once-monthly 400 mg two-injection start; HCP: healthcare professional

Figure 4 presents the most frequently cited reasons and/or barriers for HCPs opting not to use the AOM 400-TIS. The most commonly selected response was “patients don’t want two injections” (52.4%), followed by perceived issues with the safety/tolerability of the AOM 400-TIS, namely “concerns around safety of administering high dose on a single day” (34.1%) and “concerns around tolerability” (33.6%). A per-country breakdown of reasons and/or barriers for HCPs opting to not use the AOM 400-TIS is shown in Supplementary Fig. 3. Notable differences between countries were the relatively high proportion of HCPs in Germany who responded “patients don’t want two injections”, and in Sweden who responded “concerns around safety of administering high dose on a single day”.

**Fig. 5:**
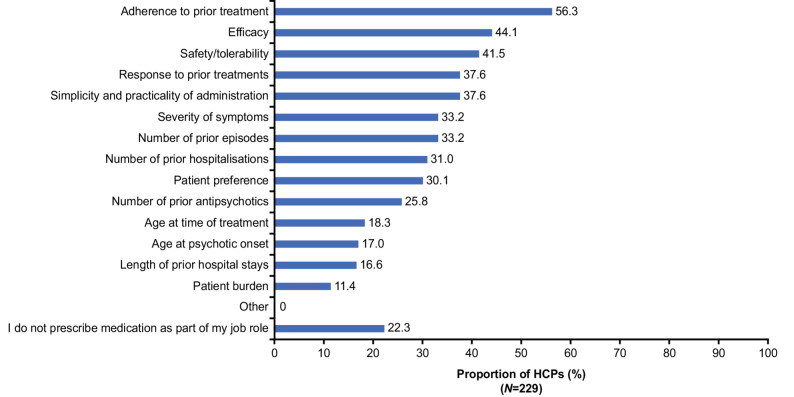
Important patient and clinical factors that influence HCPs’ decision to prescribe the AOM 400-TIS initiation regimen. Data are ranked from the most to least selected response options. Participants could select all response options that applied. AOM 400-TIS: aripiprazole once-monthly 400 mg two-injection start; HCP: healthcare professional

Figure 5 depicts patient and clinical factors that influenced HCPs’ decision to prescribe the AOM 400-TIS. The top three responses were “adherence to prior treatment” (56.3%), “efficacy” (44.1%), and “safety/tolerability” (41.5%). A breakdown of data according to country is shown in Supplementary Fig. 4. When asked to rank their responses in order of importance, HCPs in the overall cohort most often selected “adherence to prior treatment” (36.5%) and “efficacy” (12.9%) as being of greatest importance (Supplementary Fig. 5).

### Wider views and experiences with the AOM 400-TIS

**Fig. 6:**
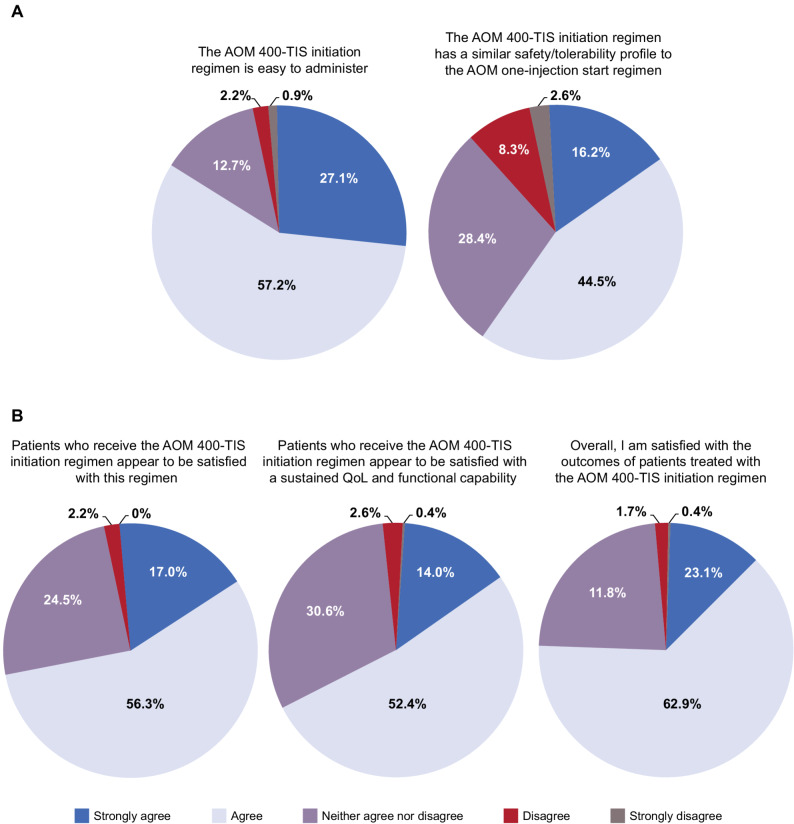
Recent HCP experience with the AOM 400-TIS initiation regimen (*N* = 229). (**A**) General. (**B**) Related to satisfaction with treatment. AOM: aripiprazole once-monthly; AOM 400-TIS: aripiprazole once-monthly 400 mg two-injection start; HCP: healthcare professional; QoL: quality of life

HCP responses to statements on their recent experience with the AOM 400-TIS, in general and related to treatment satisfaction, are shown in Fig. 6. Overall, 84.3% of HCPs agreed or strongly agreed that the AOM 400-TIS was easy to administer, while 60.7% agreed or strongly agreed that it has a similar safety/tolerability profile to an initiation regimen comprising a single injection of AOM 400 plus 14 days of oral aripiprazole supplementation. In terms of satisfaction, a majority of HCPs agreed or strongly agreed that patients appeared satisfied with the AOM 400-TIS regimen (73.4%), with 66.4% agreeing or strongly agreeing that patients appeared to be satisfied with sustained QoL and functioning following receipt of the AOM 400-TIS. Overall, 86.0% of HCPs agreed or strongly agreed that they were satisfied with the outcomes of patients who received the regimen. Participants’ responses to other statements are shown in Supplementary Fig. 6.

HCPs were asked to share any additional perceived benefits of the AOM 400-TIS in the form of open-ended responses, with major themes being: improved symptom control/outcomes; improved adherence, compliance, or treatment continuity; an early and rapid onset of action; and improved patient experience and QoL (Supplementary Table 4). HCPs were also asked to share any additional barriers or challenges they encountered with the AOM 400-TIS. Almost one-quarter of participants (22.7%) did not perceive any other barriers or obstacles to the AOM 400-TIS. Of those that did, the main theme was unwillingness on the part of the patient, for reasons such as not wanting to receive injections, not wanting to receive two injections, mistrust of the regimen, and a preference for other treatments. Tolerability/side effect concerns and cost/funding issues were also cited (Supplementary Table 5).

## Discussion

The results of the current survey, conducted in two waves over a ten-month period, offer important real-world insights into the clinical use of, and experience with, the AOM 400‑TIS initiation regimen in adults diagnosed with schizophrenia. With contributions from more than 200 HCPs, including nurses and doctors, across five European countries, and building on the preliminary first-wave findings already published [18], data show that HCPs use the AOM 400-TIS to improve treatment adherence and prevent relapse, but patient reluctance to receive two injections at the same time and concerns about the safety/tolerability of the regimen may limit its uptake.

The data reported here add to existing real-world findings on the use of the AOM 400-TIS in clinical practice in Europe. Previous studies conducted in Spain and Italy indicate that the AOM 400-TIS initiation regimen is generally well tolerated [16, 19], is perceived to have a positive impact on efficacy [19], and leads to reduced hospitalisation time and costs, when compared with a single dose of AOM plus 14 days of oral aripiprazole supplementation [19, 20]. More recent evidence on the application of the AOM 400-TIS in Europe and the surrounding region validates these earlier findings. In a small-scale Spanish study conducted in 15 patients diagnosed with schizophrenia, there were no reports of severe adverse effects [21]. Similar findings were observed in a retrospective chart review conducted at a tertiary care centre in Turkey, in which treatment initiation using the AOM 400-TIS in 29 patients with diagnoses of schizophrenia (55.2% of patients), bipolar disorder (31.0%), or schizoaffective disorder (13.8%) did not result in any adverse events leading to treatment discontinuation [22]. In addition, a clinical improvement was observed by the fifth day post-treatment in all patients except one, who showed an improvement after one week [22]. Standalone case studies in patients diagnosed with schizophrenia in Turkey also demonstrate the clinical utility of the AOM 400-TIS for the management of a patient with treatment-resistant schizophrenia with possible supersensitivity psychosis [23], and for the treatment of first-episode psychosis in a patient with human immunodeficiency virus infection receiving dual antiretroviral treatment [24].

For the most part, reasons for using and not using the AOM 400-TIS in the overall cohort of this study were similar to those reported in the first wave of the study [18]. Notably, in the current dataset, poor adherence and disease relapse were commonly cited reasons for using the AOM 400-TIS, while reluctance to receive two injections and safety/tolerability concerns were the main reasons to not use it, similar to what was observed in Wave 1 [18]. This suggests that prescriber attitudes and decision-making factors were broadly consistent across different countries/regions within Europe, and across the time periods of the two survey waves. In the overall cohort, 21.0% of HCPs cited budget constraints (i.e., lack of reimbursement) as a barrier to the use of the AOM 400-TIS, compared with a rate of 14.9% in Wave 1 [18]. This increase could reflect changes in healthcare policies or budget pressures over time between the Wave 1 and 2 survey periods and/or between-country differences in these pressures. While interesting, this finding should be interpreted with caution; although a numerical difference was evident, the data were not statistically compared and may not be significantly different.

When invited to describe in their own words any barriers or challenges associated with the AOM 400-TIS, HCPs frequently cited patient unwillingness (e.g., patients being opposed to injections in general, or opposed to receiving two injections). Since the survey did not include any patient participants — and in the absence of any published patient-level perspectives on the use of the AOM 400-TIS — it is not clear whether these reasons reflect the actual patient experience, or whether they are assumptions on the part of HCPs. This is an important distinction, since research shows that HCPs may sometimes incorrectly assume that a patient is unwilling to receive injections [25, 26]. Research also suggests that the amount of medication injected is not a primary driver of how patients and caregivers perceive LAIs; this was demonstrated in a qualitative interview study in which participants placed limited importance on the amount of medication in an injection, so long as side effects were absent or minimal [17, 27]. Whether perceived or actual, any unwillingness on the part of patients to receive the AOM 400-TIS could potentially or partly be driven by how the regimen is described/explained. For some patients, the term ‘two-injection start’ may feel invasive, and may create hesitancy, especially for those who are concerned about injections. Instead, referring to it as a ‘1‑day initiation regimen’ may feel more user-friendly and manageable to patients, by moving the focus away from the procedure itself and towards the convenience of treatment being completed at a single clinic visit without the need to continue tablet taking at home. HCPs may want to consider these differing emphases when discussing the AOM-TIS 400 initiation regimen, and tailor their language according to the concerns and preferences of the individual patient.

Consistent with the work of others [19], results from the overall cohort indicate satisfaction with the AOM 400-TIS, both on the part of HCPs and, based on their perceptions, on the part of their patients, including satisfaction related to patient QoL and functioning. Extra insight was provided when HCPs were invited to describe, in their own words (through open-ended questions), the benefits of the AOM 400-TIS, with comments such as “many patients have shown a satisfaction because of improvement of a several symptoms after having taken this [initiation] treatment” and “higher patient satisfaction”. While the current survey did not capture any data on treatment duration, it is feasible that a positive early experience with AOM 400 during treatment initiation could translate to improved adherence and persistence, in turn supporting improved long-term outcomes, including relapse prevention. Adherence data following treatment initiation with the AOM 400-TIS appear limited to three small-scale studies, but demonstrate persistence rates of ~ 75–80% after ~ 12 months [20, 28] and 66% after 24 months [21]. These findings are encouraging since adherence was an important consideration for HCPs when deciding to prescribe the AOM 400-TIS. More specifically, “poor treatment adherence” was the most common reason for initiating treatment using the AOM 400-TIS (88.6% of HCPs), “to improve adherence” was the most common goal when choosing the AOM 400‑TIS (69.4%), and “adherence to prior treatment” was the most common patient/clinical factor influencing selection of the AOM 400-TIS (56.3%; note that the survey of patient/clinical factors did not require HCPs to indicate whether this was poor or good adherence to prior treatment).

There were certain numerical differences in how the AOM 400-TIS was used in the overall cohort versus the first wave of the survey. Owing to the non-independence of the two comparison groups, such differences must be interpreted with caution, but possible explanations might include evolving practices over time and/or country-level or cultural differences in prescribing practices. For instance, there was an increase over time in the proportion of HCPs who were early adopters of the AOM 400-TIS (i.e., who had prescribed and/or administered AOM 400-TIS for more than 24 months [Wave 1, 43.6% [18]; full cohort, 55.5%]). This is not unexpected given that the second wave of the study occurred up to nine months after the first wave, allowing more time for adoption and use of the AOM 400-TIS; however, it could also be explained the inclusion of proportionately more respondents from Italy in the overall cohort, a country which, in the authors’ opinion, has demonstrated early and high rates of AOM 400-TIS adoption. Another driver appears to be the inclusion of HCPs from Nordic countries in Wave 2 of the survey, who showed the highest rates of early adoption of the AOM 400-TIS (Denmark, 64.3%; Sweden, 100%), suggesting greater confidence with the new administration option of a TIS in this region. There was also a temporal increase in the share of patients within the HCP caseload who were treated with an LAI (Wave 1, 37.2% [18]; full cohort, 45.0%), which could indicate increasing confidence with LAIs, including the AOM 400-TIS, over time. It is worth noting that LAI use in the current survey was markedly higher than what is typically observed in patients diagnosed with schizophrenia spectrum disorders in clinical practice in Europe (~ 10–20%) [29]; this likely reflects that the study inclusion criteria required that participants be experienced in prescribing and/or administering the AOM 400-TIS, indicating broader engagement with LAIs as a treatment strategy.

Overall, 16.6% of HCPs listed a “lack of clinical education” as a barrier that might lead them to not use the AOM 400-TIS. Free-text input in the survey provided additional insight on this topic, with relevant responses including “more clinical data related to its safety must be available to increase the usage”, “limited evidence based on long-term efficacy of this regime is a barrier for me”, “…clinical data from different studies related to safety and efficacy are not that available to study carefully”, and “…lack of [easily] accessible information that can be passed on to patients (leaflet, website, etc.)”. This highlights a need for targeted clinical education to improve HCP understanding of the AOM 400-TIS, including its rationale, safety profile, and practical implementation, ideally supported by accessible patient-friendly materials. Country-specific data indicate that education on the safety of the AOM 400-TIS may be particularly relevant in Sweden, where concern about administering a high dose on a single day appears to be greater relative to other countries. Similarly, prescriber education/training on the AOM 400-TIS to help increase confidence in its use may be especially relevant in Germany, where uptake of the regimen appears to be more cautious compared with other countries. In terms of safety, it should be noted that real-world evidence shows that the safety profile of the AOM 400-TIS is consistent with that for one dose of AOM [16]. Added to this, the real-world incidences of extrapyramidal adverse events with AOM are compatible with the known safety profile of AOM in clinical trials [12, 30], which is reassuring since such symptoms may be a potential hesitation to the use of AOM.

There are certain limitations of the study, in addition to those already reported [18]. Around one-half of respondents were from Italy, as extra Italian participants were recruited on a volunteer basis via the local network of the Italian member of the steering committee. This may have biased the findings towards Italian healthcare settings/treatment practices, may have skewed the results to indicate a more rapid uptake of AOM 400-TIS observed in the second wave of the study than would have been seen from a more even distribution of HCPs from the participating countries, and may limit the ability to draw pan-European conclusions. The method of recruitment (i.e., use of online panels and steering committee volunteers) may introduce systematic selection bias, while the label ‘two-injection start’ versus ‘1-day initiation’ may have influenced perceptions and uptake, and could have biased the survey responses. HCP responses were self-reported and reflective in nature, and may be based on general feelings/opinions rather than objective or systematically recorded data. Furthermore, patient perception findings are based on indirect HCP reports rather than direct patient-level data, which introduces a risk of informant bias. Lastly, because the results were purely descriptive and reported without confidence intervals, any assessment of precision/uncertainty is limited. Such limitations, however, must be balanced against the strengths of the study, including the large number of participants from multiple European countries, encompassing a variety of healthcare systems, funding models, and clinical practice settings [31].

Although specific to the AOM 400-TIS, the results reported here may provide insight into how HCPs view a TIS with Ari 2MRTU 960 (i.e., one injection of Ari 2MRTU 960, one injection of AOM 400, and a single dose of oral aripiprazole 20 mg). In the authors’ opinion, many of same drivers (e.g., poor adherence, disease relapse, patient preference) and barriers (e.g., reluctance to receive two injections, safety/tolerability concerns) could reasonably apply to a TIS with either AOM 400 or Ari 2MRTU 960. Future research in this space is warranted.

## Conclusion

The results of the current survey provide a pan-European insight into HCPs’ views and experiences of using the AOM 400-TIS in adults diagnosed with schizophrenia in real‑world practice. Improving treatment adherence and preventing relapse were the most common reasons reported by HCPs participating in the survey for prescribing AOM 400-TIS for their patients living with schizophrenia. In the opinions of the HCP participants in the surveys, patient reluctance to receive two injections and perceived safety/tolerability issues may hinder its use. Findings also indicate satisfaction with the AOM 400-TIS on the part of HCPs and, in the opinion of the participating HCPs, on the patients that they treat. Based on the HCP opinions, continued uptake of the AOM 400-TIS may be improved with increased education on the clinical evidence for its efficacy and safety, and with consideration of how the regimen is explained in patient–provider conversations.

## Supplementary Information

Supplementary Material 1.

## Data Availability

To submit inquiries related to Otsuka clinical research, or to request access to individual participant data (IPD) associated with any Otsuka clinical trial, please visit https://clinical-trials.otsuka.com/. For all approved IPD access requests, Otsuka will share anonymised IPD on a remotely accessible data sharing platform.

## Abbreviations

AOM: Aripiprazole once-monthly
AOM 400: Aripiprazole once-monthly 400 mg
AOM 400-TIS: Aripiprazole once-monthly 400 mg two-injection start
Ari 2MRTU 960: Aripiprazole 2-month ready-to-use 960 mg
HCP: Healthcare professional
LAI: Long-acting injectable
QoL: Quality of life
SD: Standard deviation
TIS: Two-injection start
UK: United Kingdom
