# Supplementary material for: Initiation of aripiprazole once-monthly via a two‑injection start in adult patients diagnosed with schizophrenia: real-world experiences of healthcare professionals from five European countries
Source: Ann Gen Psychiatry. 2026 May 31;25:66. doi: 10.1186/s12991-026-00684-z (PMC13435409; doi:10.1186/s12991-026-00684-z)
Supplement: Supplementary file 1 [file 12991_2026_684_MOESM1_ESM.docx]

## Supplementary material

# Initiation of aripiprazole once-monthly via a two‑injection start in adult patients diagnosed with schizophrenia: real-world experiences of healthcare professionals from five European countries

**Authors:** Clodagh Beckham^1*^, Murat Yildirim^2^, Andrea Fagiolini^3^, Karolina Leopold^4,5^, William J. Cottam^6^, Joe Hickey^6^, Olivia Rogerson^6*^ and Sofia Pappa^7^

**Affiliations:** ^1^Otsuka Pharmaceutical Europe Ltd., Berkshire, UK; ^2^H. Lundbeck A/S, Valby, Denmark; ^3^Università di Siena, Siena, Italy; ^4^Vivantes Hospital am Urban and Vivantes Hospital im Friedrichshain, Charite, Universitätsmedizin, Berlin, Germany; ^5^Carl Gustav Carus University Hospital, Technische Universität Dresden, Dresden, Germany; ^6^Real‑World Evidence, OPEN Health, London, UK; ^7^Division of Psychiatry, Department of Brain Sciences, Imperial College London, London, UK

*Affiliation at the time the research was conducted

**Corresponding author:** Murat Yildirim, H. Lundbeck A/S, Ottiliavej 9, Valby, 2500, Denmark; e-mail: muyi@lundbeck.com

**Supplementary Table 1.** Survey questions

| **Question/variable** | **Response options** | **Operational definitions/ comments** |  |
| --- | --- | --- | --- |
| **Questions about you** |  |  |  |
| What is your primary job role/specialty? | - Physician - General practitioner/ Primary care practitioner - Psychiatrist - Neurologist - Other (specify) - Nurse - Psychiatric - Community - Other (specify) | Categorical variable  Select one option only |  |
| Please select the setting in which you primarily work | - Specialist mental health clinic/centre - Hospital inpatient - Hospital outpatient - General practitioner - Other (specify) | Select all that apply |  |
| How many years have you worked professionally in clinical practice? | Insert number of years | Numerical variable  Response limit: 0–80 | |
| Considering your total caseload of patients (i.e., all the patients you currently treat/manage), approximately what percentage of patients have schizophrenia? | Insert percentage | Numerical variable  Response limit: 0–100 | |
| Follow-up question to above: Of your total caseload of patients that have schizophrenia, approximately what percentage of them do you treat with LAIs? | Insert percentage | Numerical variable  Response limit: 0–100 | |
| As part of your job role, are you mainly responsible for prescribing or administering the AOM 400-TIS initiation regimen to patients with schizophrenia? | - Prescribing - Administration - Both | Categorical variable  Select one option only^a^ | |

| **Questions about your current use of the AOM 400-TIS initiation regimen in the maintenance setting** | | |
| --- | --- | --- |
| Approximately how long ago did you start prescribing and/or administering the AOM 400-TIS initiation regimen to patients with schizophrenia as maintenance treatment at your treatment centre? | - Less than 6 months ago - 6–12 months ago - 13–24 months ago - More than 24 months ago | Categorical variable  Select one option only |
| Thinking about your patients who currently receive the AOM 400-TIS initiation regimen for maintenance treatment, what is the average time the patient would spend on oral aripiprazole prior to initiating this regimen? | - Up to 14 days - 14–28 days - More than 28 days | Categorical variable  Select one option only |
| On average, how severe are the symptoms of the typical patients who are initiated with the AOM 400-TIS regimen? | - Mild - Moderate - Severe | Categorical variable  Select all that apply |
| Based on your recent experience, approximately how old are the typical (adult) patients who are initiated with the AOM 400-TIS regimen at your treatment setting? | - 18–35 years - 36–64 years - 65+ years | Categorical variable  Select all that apply |
| In what treatment setting is the AOM 400-TIS typically prescribed? | - Inpatient - Outpatient - Other (specify) | Select all that apply |
| What are the most common reasons patients with schizophrenia are initiated on the AOM 400-TIS regimen as maintenance treatment after being switched from oral aripiprazole? | - Poor control of positive symptoms - Poor control of all symptoms - Relapse(s) of disease - Poor treatment adherence - Patient preference (i.e., patient not willing to take oral medication) - High rates of hospitalisations - Other (specify) | Categorical variable  Select all that apply |
| What is your main objective when choosing to prescribe AOM 400-TIS initiation regimen for maintenance treatment? | - I do not prescribe medication as part of my job role - To improve symptoms - To improve adherence - To prevent relapses - To prevent re-hospitalisations - To reduce hospital length of stay - To avoid the concomitant use of oral aripiprazole - To improve patient quality of life - To reduce burden on patients - Other (specify) | Categorical variable  Select all that apply and indicate the primary (most important) objective in your view |
| What reasons and/or barriers might lead you to not use the AOM 400-TIS initiation regimen for maintenance treatment? | - Lack of reimbursement - Lack of evidence - Patients don’t want two injections - Concerns around tolerability - Concerns around safety of administering high dose on a single day - Lack of clinical education - Other (specify) | Categorical variable  Select all that apply |
| Please indicate the most important patient and clinical factors that influence your decision to prescribe the AOM 400-TIS initiation regimen for maintenance treatment | - I do not prescribe medication as part of my job role - Age at time of treatment - Age at psychotic onset - Number of prior antipsychotics - Adherence to prior treatment - Response to prior treatments - Number of prior episodes - Number of prior hospitalisations - Length of prior hospital stays - Severity of symptoms - Efficacy - Safety/tolerability - Simplicity and practicality of administration - Patient preference - Patient burden - Other (specify) | Categorical variable  Select all that apply  Rank 1^st^, 2^nd^, and 3^rd^ most important |
| **Wave 1 only**  Aripiprazole is available as an every two-month injection of 960 mg in the USA.^b^ If the same formulation was available in Europe with the option of either a OIS (960 mg IM + 10–20 mg oral aripiprazole for 14 days) and a TIS (960 mg + AOM 400 + oral 20 mg) initiation regimen, which regimen would you choose to use? | - OIS - TIS | Categorical variable  Select all that apply |
| **Wave 2 only**  Aripiprazole is available as an every two-month injection of 960 mg in the USA and Europe. Given the option of either a OIS (960 mg IM + 10–20 mg oral aripiprazole for 14 days) or a TIS (960 mg + AOM 400 + oral 20 mg) initiation regimen, which regimen would you choose to use? | - OIS - TIS | Categorical variable  Select all that apply |

| **Questions about your wider views and experiences with the AOM 400-TIS initiation regimen** | | |
| --- | --- | --- |
| Based on your recent experience with the AOM 400-TIS initiation regimen as maintenance treatment, please indicate the extent to which you agree or disagree with the following statements: | | |
| The AOM 400-TIS initiation regimen is easy to administer | - Strongly disagree - Disagree - Neither agree nor disagree - Agree - Strongly agree | Categorical variable  Select one option |
| There is a positive impact on symptom severity during maintenance treatment in patients who are initiated with the AOM 400-TIS regimen |  |  |
| There is a positive impact on relapse frequency during maintenance treatment in patients who are initiated with the AOM 400-TIS regimen |  |  |
| Patients improve their adherence to aripiprazole treatment after AOM 400-TIS initiation regimen |  |  |
| The AOM 400-TIS regimen has a similar safety/tolerability profile to the AOM 400-OIS regimen |  |  |
| Patients who receive the AOM 400-TIS initiation regimen subsequently attend hospital less frequently than those receiving other regimens |  |  |
| The duration of hospital stays is reduced in patients who receive the AOM 400-TIS initiation regimen |  |  |
| Patients who receive the AOM 400-TIS initiation regimen appear to be satisfied with this regimen |  |  |
| Patients who receive TIS appear to be satisfied with a sustained QoL and functional capability |  |  |
| Overall, I am satisfied with the outcomes of patients treated with the AOM 400-TIS initiation regimen |  |  |
| Please indicate any other benefits in your opinion associated with the aripiprazole two-injection start regimen | Free text | Insert text in comment box |
| Please indicate any other barriers or challenges you have experienced in relation to the aripiprazole two-injection start regimen | Free text | Insert text in comment box |

^a^Participants must have selected one of the provided answers to be eligible for the survey.

^b^At the time that Wave 1 of the survey was conducted (1 February–21 March 2024 [Germany, Italy, and UK only]), an every two-month injection of aripiprazole 960 mg was approved for use in the USA, but not the European Union. The formulation was subsequently approved for use by the European Medicines Agency for the maintenance treatment of schizophrenia in adult patients stabilised with aripiprazole [1].

AOM 400: aripiprazole once-monthly 400 mg; AOM 400-OIS: aripiprazole once-monthly 400 mg one-injection start; AOM 400-TIS: aripiprazole once-monthly 400 mg two-injection start; IM: intramuscular; LAI: long-acting injectable; OIS: one-injection start; QoL: quality of life; TIS: two-injection start; UK: United Kingdom; USA: United States of America.

**Supplementary Table 2.** Characteristics of survey participants according to country

| **Characteristics** | **Denmark (*n*=28)** | **Sweden (*n*=30)** | **Italy (*n*=108)** | **United Kingdom (*n*=32)** | **Germany (*n*=31)** |
| --- | --- | --- | --- | --- | --- |
| **Primary job role/specialty, n (%)** |  |  |  |  |  |
| **Physician** |  |  |  |  |  |
| General practitioner/primary care practitioner | 0 (0) | 0 (0) | 1 (0.9) | 0 (0) | 1 (3.2) |
| Psychiatrist | 19 (67.9) | 20 (66.7) | 74 (68.5) | 20 (62.5) | 19 (61.3) |
| Neurologist | 0 (0) | 0 (0) | 0 (0) | 0 (0) | 0 (0) |
| Other^a^ | 1 (3.6) | 0 (0) | 2 (1.9) | 0 (0) | 0 (0) |
| **Nurse** |  |  |  |  |  |
| Psychiatric | 8 (28.6) | 10 (33.3) | 25 (23.1) | 10 (31.3) | 9 (29.0) |
| Community | 0 (0) | 0 (0) | 4 (3.7) | 2 (6.3) | 2 (6.5) |
| Other^b^ | 0 (0) | 0 (0) | 2 (1.9) | 0 (0) | 0 (0) |
| **Primary work setting, n (%)^c^** |  |  |  |  |  |
| Specialist mental health clinic/centre | 17 (60.7) | 19 (63.3) | 83 (76.9) | 22 (68.8) | 11 (35.5) |
| Hospital inpatient | 18 (64.3) | 25 (83.3) | 28 (25.9) | 10 (31.3) | 13 (41.9) |
| Hospital outpatient | 19 (67.9) | 18 (60.0) | 14 (13.0) | 7 (21.9) | 11 (35.5) |
| General practitioner | 7 (25.0) | 4 (13.3) | 3 (2.8) | 0 (0) | 5 (16.1) |
| Other^d^ | 0 (0) | 0 (0) | 6 (5.6) | 1 (3.1) | 2 (6.5) |
| **Time working professionally in clinical practice, median (IQR) [range], years** | 15.0 (10.0–24.3) [3–42] | 15.5 (11.3–19.8) [7–25] | 20.0 (10.0–29.3) [1–69] | 21.5 (12.3–28.3) [3–53] | 22.0 (16.0–25.0) [8–35] |
| **Proportion of patients in caseload diagnosed with schizophrenia, median (IQR) [range], %** | 25.0 (15.0–30.0) [10–90] | 16.0 (15.0–23.8) [5–100] | 30.0 (20.0–46.3) [3–100] | 52.5 (40.0–71.3) [5–95] | 25.0 (15.0–32.5) [5–100] |
| **Proportion of patients in caseload diagnosed with schizophrenia and treated with LAIs, median (IQR) [range], %** | 57.5 (50.0–65.0) [3–75] | 65.0 (50.0–70.0) [40–90] | 40.0 (20.0–65.0) [2–100] | 50.0 (28.8–56.3) [0–100] | 25.0 (20.0–40.0) [5–75] |
| **Main responsibility regarding the AOM 400-TIS, n (%)** | | | | | |
| Prescribing | 10 (35.7) | 16 (53.3) | 50 (46.3) | 20 (62.5) | 8 (25.8) |
| Administration | 8 (28.6) | 9 (30.0) | 27 (25.0) | 8 (25.0) | 9 (29.0) |
| Both | 10 (35.7) | 5 (16.7) | 31 (28.7) | 4 (12.5) | 14 (45.2) |

^a^Other responses included: medical resident (*n*=1) [Italy]; psychiatry resident (*n*=1) [Italy]; and physician under training in psychiatry (*n*=1) [Denmark].

^b^Other responses included: ward physician (*n*=1) [Italy]; and hospital (*n*=1) [Italy].

^c^Participants could select all response options that applied.

^d^Other responses included: ‘provide specialist second opinions for treatment’ (*n*=1) [United Kingdom]; ‘psychiatric practice’ (*n*=1) [Germany]; ‘specialist practice’ (*n*=1) [Germany]; ‘private clinic’ (*n*=1) [Italy]; ‘mental health centre’ (*n*=1) [Italy]; ‘outpatient clinic, rehabilitation facilities’ (*n*=1) [Italy]; ‘psychiatric diagnosis and treatment unit (Servizio Psichiatrico di Diagnosi e Cura)’^e^ (*n*=1) [Italy]; ‘psychosocial centre’ (*n*=1) [Italy]; and ‘university psychiatry department’ (*n*=1) [Italy].

^e^Servizio Psichiatrico di Diagnosi e Cura is an Italian hospital unit where voluntary and mandatory inpatient psychiatric treatment is provided; it is based within hospital facilities and it is part of the mental health department.

AOM 400-TIS: aripiprazole once-monthly 400 mg two-injection start; IQR: interquartile range; LAI: long-acting injection.

**Supplementary Table 3**. HCP use of the AOM 400-TIS initiation regimen in a maintenance treatment setting according to country

|  | **Denmark (*n*=28)** | **Sweden (*n*=30)** | **Italy (*n*=108)** | **United Kingdom (*n*=32)** | **Germany (*n*=31)** |
| --- | --- | --- | --- | --- | --- |
| **Approximate time since HCP started to prescribe/administer the AOM 400-TIS, n (%)** | | | | | |
| <6 months ago | 4 (14.3) | 0 (0) | 15 (13.9) | 3 (9.4) | 5 (16.1) |
| 6–12 months ago | 4 (14.3) | 0 (0) | 17 (15.7) | 5 (15.6) | 9 (29.0) |
| 13–24 months ago | 2 (7.1) | 0 (0) | 27 (25.0) | 5 (15.6) | 6 (19.4) |
| >24 months ago | 18 (64.3) | 30 (100) | 49 (45.4) | 19 (59.4) | 11 (35.5) |
| **Average time spent by patient on oral aripiprazole prior to initiating AOM via the AOM 400-TIS, n (%)** | | | | | |
| <14 days | 11 (39.3) | 12 (40.0) | 50 (46.3) | 12 (37.5) | 2 (6.5) |
| 14–28 days | 11 (39.3) | 18 (60.0) | 41 (38.0) | 9 (28.1) | 14 (45.2) |
| >28 days | 6 (21.4) | 0 (0) | 17 (15.7) | 11 (34.4) | 15 (48.4) |
| **Average severity of symptoms of typical patients whose treatment with AOM was initiated via the AOM 400-TIS, n (%)^a^** | | | | | |
| Mild | 4 (14.3) | 6 (20.0) | 25 (23.1) | 4 (12.5) | 4 (12.9) |
| Moderate | 14 (50.0) | 22 (73.3) | 74 (68.5) | 21 (65.6) | 19 (61.3) |
| Severe | 21 (75.0) | 26 (86.7) | 40 (37.0) | 13 (40.6) | 17 (54.8) |
| **Age of typical (adult) patients whose treatment with AOM was initiated via the AOM 400-TIS, n (%)^a^** | | | | | |
| 18–35 years | 27 (96.4) | 28 (93.3) | 73 (67.6) | 14 (43.8) | 20 (64.5) |
| 36–64 years | 12 (42.9) | 16 (53.3) | 75 (69.4) | 22 (68.8) | 23 (74.2) |
| ≥65 years | 0 (0) | 0 (0) | 6 (5.6) | 4 (12.5) | 5 (16.1) |
| **Treatment setting in which the AOM 400-TIS is typically prescribed, n (%)^a^** | | | | | |
| Inpatient | 21 (75.0) | 28 (93.3) | 64 (59.3) | 20 (62.5) | 17 (54.8) |
| Outpatient | 12 (42.9) | 12 (40.0) | 74 (68.5) | 24 (75.0) | 22 (71.0) |
| Other^b,c^ | 1 (3.6) | 0 (0) | 2 (2.6) | – | – |

^a^Participants could select all response options that applied.

^b^This response option was only included in Wave 2 of the survey.

^c^Other responses included: ‘both’ (*n*=2) [Italy]; and ‘a constant carer is available’ (*n*=1) [Denmark].

AOM: aripiprazole once-monthly; AOM 400-TIS: aripiprazole once-monthly 400 mg two-injection start; HCP: healthcare professional.

**Supplementary Table 4.** HCP free-text responses to the question ‘Please indicate any other benefits in your opinion associated with the aripiprazole two-injection start regimen’

| **Theme** | **HCP responses** |
| --- | --- |
| **Improved symptom control/outcomes (*n*=46)** | - **It works well in terms of symptom control** with less metabolic side effects - Better control of positive symptoms - Tolerability, low side effects, **improved relapse periods** - Effectiveness, alternative, hope for better functional level - Excellent outcomes - Fewer relapses - Faster onset of action, **better efficacy** - User-friendliness, **efficacy** - Improved efficacy - **Longer response to therapy**, less effort for the patient and for us as staff - Better adherence, **better efficacy** generally speaking - Patient has fewer doctor’s visits, thus better quality of life; **rapid and long-lasting efficacy**, we have less work, compliance is promoted, no need to check whether medication is taken or not - Better control of negative symptoms - Fewer recurrences - Greater control of patient symptoms - Effective after a few treatments - Efficacy - Efficacy - Certainty of efficacy - No side effects, **cognitive and emotional improvement**, recovery of quality of life, no sexual issues, improved empathy - Increased efficacy - Patients receiving AOM-TIS have fewer relapses than patients who had received single doses - Stability - Improved efficacy - The work that is done on the **symptoms** is the biggest advantage in my opinion - With this treatment the patients have achieved **improved results in the form of symptom relief** and therefore their quality of life has also improved - It can improve the patient’s general well-being by having a large impact on the reduction of the number of seizures - Increased compliance to the treatment, **symptom management** and easy administration are some of the biggest advantages in my opinion - Different advantages with this treatment includes the efficacy in shorter time, fewer hospitalisations and **better symptom improvement** - It helps with **reducing symptoms** and makes it possible for the patients to go back to their normal routine, which improves their overall quality of life - Symptoms of schizophrenia can be significantly reduced with this regime that needs to be administered once a month - it can help patients to **gradually improve their symptoms** and it also reduces the need of taking it on daily basis since the injection is once a month - Many patients have shown a satisfaction because of improvement of a several symptoms after having taken this treatment - Aripiprazole can manage symptoms more efficiently than other treatments, which makes it a valuable alternative for patients who are managing schizophrenia - This drug helps in improving the condition in patients with Schizophrenia, which leads to **better management of symptoms** and improved quality of life - We will avoid medicine failure with this regime - Aripiprazole double injection can be a good support for patients with poor adherence as it reduces the need of daily medication and ensures more **consistent therapeutic effects** - It has a **positive impact on symptoms**, which results in better function and increased quality of life - Aripiprazole double injection also helps in achieving better therapeutic results with their double injection regime - Since it is a double injection regime it could be more efficient […] giving **sustained symptom control** and at the same time minimise the frequency of administration for patients - It helps in giving a better quality of life by **reducing symptoms** and give the patients the possibility to engage fully in their personal and social activities - It could help in **reducing the risk for relapse** by maintaining stable medicine levels in the bloodstream, which helps the **continuously symptom management** and the overall treatment continuity - High safety, **symptom remission**, and faster patient stabilisation. Performing the first vial also allows assessment of tolerability to the LAI formulation - Handling, safety, **efficacy**, tolerability, and ability to adapt to the preferences and quality of life not only in the short term but also in the long term of our patients - Adherence to therapy and **reduced recurrence** - Adherence, tolerability, **effectiveness in symptom control** |
| **Improved adherence/ compliance/treatment continuity (*n*=42)** | - Removes the risk of non-adherence with oral medication - Easy start, **ensuring compliance** in patients that previously responded to aripiprazole - Avoids concerns about oral adherence - It can be difficult to ensure adherence to the oral doses when using the OIS initiation. The two-injection initiation eliminates this problem - Reduction in admissions and **improved compliance with treatment** - Knowing patient is compliant - Improves adherence in the initial phase of long-acting treatment, showing benefits to the patient and eventually making it possible to work on patient compliance in general (service attachment, rehabilitation projects) - High compliance - Safe start of medication with fluctuating compliance - **Better adherence**, better efficacy generally speaking - **Therapy adherence** and tolerability - Patient has fewer doctor’s visits, thus better quality of life; rapid and long-lasting efficacy, we have less work, **compliance is promoted, no need to check whether medication is taken or not** - Treatment adherence through 2 visits - Healthcare provider confidence in medication intake - Improved adherence - Improved adherence - Better compliance in general - Improved compliance - Adherence - Immediate adherence - Improved patient compliance - With noncompliant patients to oral administration, it allows better adherence - Quicker efficacy, shorter hospital stays, **increased compliance**, less side effects and good tolerability - It works best for patients with high or no compliance - Some of the most important advantages with this regime is the rapid symptom control and the **improved compliance towards the treatment** - Initiation regime with double injections gives results quicker which makes it **easier for the patients to follow the routine** - Patients often forget to take the oral medications, in those cases this regime will be more comfortable for them to follow - **Increased compliance to the treatment**, symptom management and easy administration are some of the biggest advantages in my opinion - There are **smaller problems with the compliance** of this scheme since it is easy to remember compared to others. - It can efficiently **improve the patient’s compliance of treatment routines** since it is easy to follow and avoids the risk of forgetting about the routine - It is very beneficial for patients who have a history of not following the treatment alternatives - **Secure compliance**, weight neutral - Aripiprazole double injection can be a **good support for patients with poor adherence** as it reduces the need of daily medication and ensures more consistent therapeutic effects - It could help in reducing the risk for relapse by maintaining stable medicine levels in the bloodstream, which helps the continuously symptom management and the **overall treatment continuity** - Aripiprazole is a monthly injection which could be practical for the patients, which gives them the possibility to not be worried about their treatments and **reduces the likelihood of skipping and missing doses** - Assured adherence - Promptness of treatment and **patient compliance** - Increased adherence - Mainly beneficial on ease of therapy management and **adherence** - Better adherence to therapy - **Adherence to therapy** and reduced recurrence - **Adherence**, tolerability, effectiveness in symptom control |
| **Early/rapid onset of action (*n*=32)** | - Better and quicker loading bolus of aripiprazole - Reduced hospital length of stay, **chance for quicker initiation of treatment** even under emergency conditions - Fast saturation, fast effectiveness - Fast saturation - Reduction of symptomatology in a short period of time - **Faster onset of action**, better efficacy - Patient has fewer doctor’s visits, thus better quality of life; **rapid and long-lasting efficacy**, we have less work, compliance is promoted, no need to check whether medication is taken or not - Faster - Less time to coverage, fewer cases of insufficient coverage in the acute phase - Rapid symptomatic remission - Rapid symptom control, especially for patients with extremely short treatment durations who are admitted in a protected manner, and therefore better nursing care less risk to patients and staff - Pharmacokinetic latency - Early onset of effective long-term treatment - In my opinion, the 400-TIS has faster onset of action - **Quick induction time**, reduced hospital stay time - Reduced response time - Increased speed in reaching steady state and relative clinical efficacy (with just a single administration, full psychopharmacological response was often seen between the X and 6^th^ month of LAI administration) - Quickness of action - Very early positive effects compared with other medication types - Quicker efficacy - **Quicker efficacy**, shorter hospital stays, increased compliance, less side effects and good tolerability - it offers the quickest stabilisation which is a very important factor for many patients - Some of the most important advantages with this regime is the **rapid symptom control** and the improved compliance towards the treatment - Initiation regime with double injections **gives results quicker** which makes it easier for the patients to follow the routine - For frequent episodes of schizophrenia it is important that the treatment is efficient and quick, I think that double injection method would be suitable for that - With this the patients can **get results in shorter period of time** and would also lead to improvement in their quality of life - Different advantages with this treatment includes the **efficacy in shorter time**, fewer hospitalisations and better symptom improvement - This treatment gives quicker symptom control compared to others, so it works for many patients - Administration is more efficient than others, since it is given intravenously, which enables quick absorption and instant therapeutic effects - High safety, symptom remission, and **faster patient stabilisation**. Performing the first vial also allows assessment of tolerability to the LAI formulation - Speed of action - **Promptness of treatment** and patient compliance |
| **Improved patient experience and quality of life (*n*=28)** | - [Stops] you having to distress the patient by having to insist on oral meds too - Easy interaction time with the clients - Patient-friendly approach - **User-friendliness**, efficacy - Psychological well-being - Peace of mind - Patient has fewer doctor’s visits, thus **better quality of life**; rapid and long-lasting efficacy, we have less work, compliance is promoted, no need to check whether medication is taken or not - Improved patient’s quality of life - Patient’s preference - I believe the aripiprazole two-injection has transformed many patients’ lives. It has reduced patients’ admissions to hospital and has **improved their quality of life**. It has been a revolutionary process for many patients - **Higher patient satisfaction** and greater safety for caregivers - No side effects, cognitive and emotional improvement, **recovery of quality of life**, no sexual issues, improved empathy - Anticipated positive effect - Benefit in the patient's life - With this treatment the patients have achieved improved results in the form of symptom relief and therefore their **quality of life has also improved** - Since it can be taken by younger patients it can work as a good alternative to improve their quality of life earlier - With this the patients can get results in shorter period of time and would also lead to **improvement in their quality of life** - It helps with reducing symptoms and makes it **possible for the patients to go back to their normal routine, which improves their overall quality of life** - This drug helps in improving the condition in patients with Schizophrenia, which leads to better management of symptoms and **improved quality of life** - Easy administration, **quality of life** - With this treatment the patients can go back to their daily activities quicker, which improves their quality of life - Young patients that tolerates it well, becomes productive very early and improves their life significantly - It helps the patient to be more active in their daily life, which enhance a better physical healing and increases the mental well-being - It has a positive impact on symptoms, which results in better function and increased quality of life - It helps in **giving a better quality of life** by reducing symptoms and give the patients the possibility to engage fully in their personal and social activities - Aripiprazole is a monthly injection which could be practical for the patients, which **gives them the possibility to not be worried about their treatments** and reduces the likelihood of skipping and missing doses - Handling, safety, efficacy, tolerability, and **ability to adapt to the preferences and quality of life** not only in the short term but also in the long term of our patients - **Quality of life** and management of family relationships |
| **Good tolerability/safety profile (*n*=27)** | - It works well in terms of symptom control with **less metabolic side effects** - Generally well tolerated - Better tolerability in terms of extrapyramidal symptoms - **Tolerability, low side effects**, improved relapse periods - Patients are more likely to tolerate medication with fewer negative side effects. Educating and informing patients is an important part of preparation for commencement of the treatment - Low side effect profile - Therapy adherence and **tolerability** - Offers an option that is safer in those with cardiac history - More verifiable tolerability - Tolerability - Safe to use in patients with CVD, good [side effect] profile - Less drowsiness - Less side effects - Relative quietness about adverse events - Excellent tolerability - **No side effects**, cognitive and emotional improvement, recovery of quality of life, **no sexual issues**, improved empathy - Good drug, makes it easier for treatment within forensic psychiatry, **good metabolic control**, patient trust the medicine - Quicker efficacy, shorter hospital stays, increased compliance, **less side effects and good tolerability** - There are no severe side effects connected with this treatment regime - Secure compliance, **weight neutral** - Less sedation - Increased safety in the immediate post-discharge period - Good tolerability of treatment - **High safety**, symptom remission, and faster patient stabilisation. **Performing the first vial also allows assessment of tolerability to the LAI formulation** - Handling, **safety**, efficacy, **tolerability**, and ability to adapt to the preferences and quality of life not only in the short term but also in the long term of our patients - Safety - Adherence, **tolerability**, effectiveness in symptom control |
| **Simple/convenient (n=24)** | - Does not need post injection monitoring that LAI olanzapine needs - Simpler for patients, less chance of confusion about starting regimen - **Easy start**, ensuring compliance in patients that previously responded to aripiprazole - Convenient - Very convenient and simple initial treatment - Practicality - Simplicity of treatment management - Patients don’t require oral medication - Reduced oral co-administration period - Overall easier - Easy to prescribe - Convenience - Avoid oral aripiprazole right away - You avoid both injection and pills - Increased compliance to the treatment, symptom management and **easy administration** are some of the biggest advantages in my opinion - It can help patients to gradually improve their symptoms and it also **reduces the need of taking it on daily basis** since the injection is once a month - Convenient concept - **Easy**, **practical** and efficient - **Easy administration**, quality of life - The patients are pleased, as they do not have to worry about lack of doses and that stress that comes along with that - Absolutely no need for oral medication compared to other treatments is a big advantage - The administration could be more comfortable for patients, since it involves monthly injections rather than daily oral medication, which is very good for those with a busy lifestyle - Absence of [oral] therapy - Mainly beneficial on ease of therapy management and adherence - Convenience |
| **Improved efficiency and time management for HCPs and patients (*n*=9)** | - Longer response to therapy, **less effort for the patient and for us as staff** - **Patient has fewer doctor’s visits**, thus better quality of life; rapid and long-lasting efficacy, **we have less work**, compliance is promoted, no need to check whether medication is taken or not - **Less effort, more time for the patient or for others**, better level of active ingredient - I don’t see any benefits here apart from having to spend less time - Patient has to go to the doctor less often, he and we have to sacrifice less time as visits are less frequent - Number of patient contacts would be reduced - Less strain on the healthcare - Easy, practical and **efficient** - Since it is a double injection regime it could be **more efficient** […] giving sustained symptom control and at the same time **minimise the frequency of administration for patients** |
| **Reduced hospitalisation/ re-hospitalisation (*n*=12)** | - **Reduction in admissions** and improved compliance with treatment - Reduced rate of re-hospitalisation - **Reduced hospital length of stay**, chance for quicker initiation of treatment even under emergency conditions - I believe the aripiprazole two-injection has transformed many patients’ lives. **It has reduced patients’ admissions to hospital** and has improved their quality of life. It has been a revolutionary process for many patients - Length of hospital stay - Quick induction time, **reduced hospital stay time** - A chance to discharge the patient earlier from the ward - Faster discharge - Quicker efficacy, **shorter hospital stays**, increased compliance, less side effects and good tolerability - This can reduce the length of the hospital stays, which again reduces the total cost for the treatment for the patients - Different advantages with this treatment includes the efficacy in shorter time, **fewer hospitalisations** and better symptom improvement - Quicker discharge from hospital with need for observation of intake of pills for i.e. convicted patients |
| **Stable plasma aripiprazole levels (*n*=3)** | - Achievement of stable level of active ingredient over 24h - More constant plasma levels - It could help in reducing the risk for relapse by **maintaining stable medicine levels in the bloodstream**, which helps the continuously symptom management and the overall treatment continuity |
| **Other (*n*=24)** | - One of the cleanest LAIs - Patients refuse oral medication - Regular contact with a mental health professional - Regular visits improve engagement with teams - Frequency - Better understanding of the mental health difficulties to maintain a level of stability - Manageability - Modularity in intramuscular drug intake - Initially patients are often easier to convince - The benefit of it being a partial agonist - 2 consultations - Less effort, more time for the patient or for others, better level of active ingredient - Tolerability monitoring - Higher patient satisfaction and **greater safety for caregivers** - Effective even on concomitant affective symptoms (schizoaffective syndrome) - No side effects, cognitive and emotional improvement, recovery of quality of life, no sexual issues, **improved empathy** - More peace of mind for caregivers - Be helped by the regional body - **Good drug**, **makes it easier for treatment within forensic psychiatry**, good metabolic control, **patient trust the medicine** - This regime involves a high risk and high award, and works very well for your patients - You do not have to give it gluteal - Works for most - When the patient is hospitalised it is very easy to implement this treatment, because we are monitoring them and manage their symptoms and side effects - **Handling**, safety efficacy, tolerability, and ability to adapt to the preferences and quality of life not only in the short term but also in the long term of our patients |
| **No additional benefits identified/supplied (*n*=36)** | - N/A - Not sure - All the benefits AOM400-TIS regimen brings to the patient’s quality of life have already been listed above - No problems to comment on - No additional benefits - None - No benefit in percent compared with initial injection alone - No other - I actually don’t see any - No additional - None given - [The main advantages … have already been mentioned] - No additional comments - I don't see any other benefits - I don't know - None - . - ..... - ... - It is all already outlined - [Left blank] - I have already pointed out my opinion - I think we have described them all - N/A - All are mentioned - – - . - No other - None - None - . - . - . - Don't know - None - No comments |

Free-text responses were received in the participants’ local language and were translated into English where required.

Participant responses were grouped into common themes, shown in order from themes with the most to the least number of relevant responses (excluding the ‘Other’ and ‘No additional benefits identified/supplied’ themes, which are listed last).

Responses may have encompassed multiple themes; in such cases, they were included in more than one category (text shown in bold refers to the portion of a response that is relevant to the selected category).

AOM 400-TIS: aripiprazole once monthly 400 mg two-injection start; CVD: cardiovascular disease; HCP: healthcare professional; LAI: long-acting injectable; N/A: not applicable; OIS: one-injection start.

**Supplementary Table 5.** HCP free-text responses to the question ‘Please indicate any other barriers or challenges you have experienced in relation to the aripiprazole two-injection start regimen’

| **Theme** |  | **HCP responses** |
| --- | --- | --- |
| **Patient unwilling (*n*=69)** | **General reluctance / refusal (*n*=20)** | - Agreement to begin treatment - A very small number of patients have not accepted the TIS regimen, but this also happens with other treatment regimens and is part of the regular patient management - Persuading patients to participate - Low patient preference - Acceptance from patient - **Patients reject it**, reimbursement is questionable, too much active ingredient in the body - Compliance with 2 injections, **patient’s fear** - Uncooperative patient - Patient’s refusal - Patient’s refusal - Acceptability - Persuading the patient - Patient acceptance - Indication, **patient opposition**, costs - Patient refusal - Patient opposition - Patient non-acceptance - Patient refuses - Some patients does not want it - Patient concerns |
|  | **Opposed to injections (*n*=9)** | - Patients not liking injections - Patients not willing to have injections - Patient aversion to injections - Injections are unpopular - Injections are not popular with patients - **Fear of needles** and potential side effects make some patients worried - The patient does not want injection treatment - **Resistant towards depot**, price - **Not all patients are ready to take injections**, some might have anxiety receiving them, which could make compliance to the treatment plan difficult |
|  | **Opposed to two injections (*n*=31)** | - Having 2 injections at once, patients not keen at times - **Concerns about multiple injections** and tolerability - Patient not always wanting x2 injections - **Patients may dislike the two injections** or show concern about tolerability - Patients dislike the two injections - Patient rejects 2 injections - Patients accepting 2 injections - Some patients don’t want the 2 injections, otherwise there are no obstacles - 2 injections could be a problem, but I don’t see any concerns there - **Most people reject 2 injections in a row**. And what happens if too much of the active ingredient remains in the body? What are the reactions to this? - Patients don’t want 2 injections - It could be a problem cost-wise, **patients may not want 2 injections** - Patients often don’t accept 2 injections. Even a continuous injection system requires a lot of convincing - Patients tend to be sceptical about 2 injections, 1 should be enough, they wouldn’t be that seriously ill after all - None outside of patient preference for 1 injection only - Patients don’t want 2 injections in one day - Some difficulty in offering the user and family members double dosing. However, this is easily overcome with proper information - Patient interpretation over performing two injections - Slight opposition from the patient to undergo double injection - Sometimes **patients refuse double injection**, and other times oral administration is preferred in order to verify tolerability - Some patients does not want double injections - **Patients’ unwillingness to double injections**, possibly increased side effects? Akathisia? - Some of the patients might have problems with taking the two injections, which can result in discomfort for them - To switch from oral medication to double injections would be difficult for some of the patients, which makes it a challenge for them - To have two injections at the time could be a bit complicated for those patients who feel nervous when receiving injections - Few patients feel worried as the believe they have to take more injections during one day - The need that occurs after having taken this double injection is often challenging for some patients - **Uneasy about nurse who has to administer 2 depot injections**. Worries about side effects of akathisia. Worries about allergic reaction - Double initiation - Two injections - Injections at two different sites |
|  | **Mistrust (*n*=6)** | - Patient mistrust - **Patient distrust**, fear of too much sedation - Distrust of some patients to the adherence of traditional therapies - Some patients, especially early in their treatment with high severity is a challenge to administer because of paranoia related to more needles - To convince the patients about a complete injection treatment and win trust is sometimes a challenge - Patient preference could be a challenge, **if the patient does not trust the treatment**, as this discomfort can result in lack of compliance and negative impact on treatment results |
|  | **Preference for other treatments (*n*=3)** | - Sometimes patients refuse double injection, and other times **oral administration is preferred in order to verify tolerability** - If there are not many oral doses that are missed many **patients prefer other injection treatments** in order to manage side effects - **Patient preference could be a challenge**, if the patient does not trust the treatment, as this discomfort can result in lack of compliance and negative impact on treatment results |
| **Tolerability/side effect concerns  (*n*=36)** | | - Concerns about multiple injections and **tolerability** - Patients that do not respond to aripiprazole. **Worries about increases side effects** and lack of [easily] accessible information that can be passed on to patients (leaflet, website etc.) - [Extrapyramidal side effects] - Patient tolerability - More likely to expect [extrapyramidal symptoms] - Some fatigue or intolerance events - Patients may dislike the two injections or **show concern about tolerability** - Individual cases with [adverse drug reactions] - Most people reject 2 injections in a row. And what happens if too much of the active ingredient remains in the body? **What are the reactions to this?** - Onset of akathisia - Increased frequency of extrapyramidal symptoms - Fear of poor individual tolerability - Side effects - Sometimes double administration results in **sedation** and there is significant pharmacological constraints compared with single administration - Patient distrust, fear of too much **sedation** - Tolerability. 23-year-old patient with severe muscle stiffness after TIS administration, currently hospitalised for that reason - Observed nausea at times - Patients’ unwillingness to double injections, **possibly increased side effects**? **Akathisia**? - **Tolerability**, **safety** and a lot of monitoring are the most important factors to why this might not suit all - Fear of needles and **potential side effects** make some patients worried - Worry related to this methods safety could be a barrier for some patients and healthcare personnel - It could be a challenge for patients who do not tolerate the treatment - I think the associated side effects of this treatment method would be a barrier for many - It has some serious side effects which could be a challenge for the patient to follow the treatment plan and sustain their overall well-being - Uneasy about nurse who has to administer 2 depot injections. **Worries about side effects of akathisia**. **Worries about allergic reaction** - Akinesia - When you initiate a tear **treatment tolerability** and symptom management is a challenge because of a high dose given during one single day - Elderly patients need more monitoring in order to control potential damaging side effects - Since it is a double injection regime with 400 mg it can be damaging for the patient, if their body cannot tolerate it - It has the highest mg level which could be beneficial to achieve therapeutic effects, but it could also increase the risk of side effects if it is not managed correctly - Patients can experience side effects after having received the injection which could lead to them rejecting the treatment, and in the end it will have an impact on their general health and healing - This medication have a few serious side effects that can be dangerous, which means you need to contact the doctor. If this occurs then it will be a challenge for the patients to control their treatment and keep their trust in the treatment - Aripiprazole could be damaging to some patients with low tolerability, because its high dosage at administration could potentially lead to **increased side effects or side effects that requires close monitoring and adjustment** - Uncommon side effects but impair continued therapy : Akathisia - Safety - Tolerability risk |
| **Cost and funding issues  (*n*=24)** | | - Pricing may make it difficult for some primary care teams to take over prescribing and administration - Cost - Resistance due to cost - Patients reject it, **reimbursement is questionable**, too much active ingredient in the body - **It could be a problem cost-wise**, patients may not want 2 injections - Costs - Funding - in my NHS workplace we have to gain approval from our head pharmacist on a case by case basis - Costs - High costs - Indication, patient opposition, **costs** - Economic - We hope for a good price for the medication - The costs for the whole treatment could be a challenge for many patients who comes to our clinic - The cost could be a challenge for some who do not have a high coverage - The **cost of it** and the availability could be a barrier for many patients who cannot afford the treatment - The overall treatment could be costly for some patients, which makes it a barrier for them to follow the schedule - Resistant towards depot, **price** - The overall costs of the treatment has been a barrier for some patients - It can affect a patient’s economy as it can be **expensive for some**, which can potentially result difficulties getting access to treatment or be compliant to their medication - The cost of the medicine could be a barrier, especially for patients who do not have good insurance coverage - **Costs** and supplies - **Cost** and eventual availability in settings more difficult for our patients to reach - Economic budget constraints - Cost |
| **Gaps in information, education, or training/experience (*n*=15)** | | - Fear from the nurses - Patients that do not respond to aripiprazole. Worries about increases side effects and **lack of [easily] accessible information that can be passed on to patients (leaflet, website etc.**) - Never tested - Not a lot of information to hand - Explanation of rationale for double injection in suspicious or ambivalent patients - Lack of experience - Poorly trained nurses feared administering two vials - The challenge is to gain more experience - Administration of two injections can be a bit challenging since it requires well educated personnel in order to avoid pain and discomfort for the patients - More clinical data related to its safety must be available to increase the usage - Limited evidence based on long-term efficacy of this regime is a barrier for me - In my opinion the clinical data from different studies related to safety and efficacy are not that that available to study carefully - Education about tolerable doses considering worries about side effects - The patients should already have taken the necessary precautions before taking it, so it could be a challenge to ensure that they are fully prepared and informed about potential risks and side effects - Getting the patient to understand the treatment strategy |
| **Concerns about dosage/drug accumulation (*n*=14)** | | - Can sometimes be perceived as high dosage by the patient - Most people reject 2 injections in a row. And **what happens if too much of the active ingredient remains in the body**? What are the reactions to this? - Problem is, if the drug is injected, then it is a problem to get it out of the body, so to speak, when it would be indicated or necessary - Patients reject it, reimbursement is questionable, **too much active ingredient in the body** - You need to take precautions when you take this drug as it has a high dosage - Patients could be worried about the high dosage of the injection, which could be challenging for them to manage and could have an impact on their willingness to follow the treatment - Since it has a higher dosage in its regime, patients might feel doubt or trust question about the treatment’s safety and efficacy, which can have an impact on their willingness to follow the prescribed plan - High dosages - Worry. **High dosage** - Uncertainty with patient, afraid of too high doses right away - When you initiate a tear treatment tolerability and symptom management is a challenge because of a high dose given during one single day - Patients can be worried about the safety of taking high doses in one single day, which could be a barrier for their treatment as it can result in hesitation or rejection to participating in the treatment - Aripiprazole could be damaging to some patients with low tolerability, because its **high dosage at administration** could potentially lead to increased side effects or side effects that requires close monitoring and adjustment - Fear of overdosing |
| **Formulary and availability issues  (*n*=11)** | | - Getting on local formulary - Not on formulary - Sometimes it isn’t [on the] formulary, so isn’t available in the pharmacy - Immediate drug availability as an order takes time to be distributed to the centre - Difficult to bring in something new - Availability from hospital pharmacy - I believe the availability of this treatment alternative needs to be improved in order for it to be used more and to a greater extent - The cost of it and the **availability** could be a barrier for many patients who cannot afford the treatment - It can affect a patient’s economy as it can be expensive for some, which can potentially result **difficulties getting access** to treatment or be compliant to their medication - Costs and **supplies** - Cost and **eventual availability** in settings more difficult for our patients to reach |
| **Logistical, scheduling, or staff-related challenges (*n*=11)** | | - Patient’s unavailability - Organisation - Availability of times and days for administration - Insufficient nursing staff - Bureaucracy - Impractical - Staff concerns - Some colleagues' opposition - Patient's availability - Tolerability, safety and **a lot of monitoring** are the most important factors to why this might not suit all - To monitor the patients during initiation of this scheme in a hectical scheme would increase our work |
| **Adherence and compliance concerns  (*n*=7)** | | - Patient’s compliance - Lower compliance - Patient adherence - Poor adherence - **Compliance with 2 injections**, patient’s fear - Patient compliance - Risk of [non-adherence] to therapy |
| **Other (*n*=17)** | | - **Patients that do not respond to aripiprazole**. Worries about increases side effects and lack of [easily] accessible information that can be passed on to patients (leaflet, website etc.) - If a patient is particularly unwell, it can [be] difficult to administer two separate injections - Reluctance from colleagues - Convenience - Slight difficulty - There have been patients who the 400mg dose has not been effective so they require oral aripiprazole tablets as top ups - Concerns patient, relatives, younger colleagues. Necessary persuasion - Improves symptoms - Family stigma - Resistance to 3 vials - **Indication**, patient opposition, costs - Difficulty explaining it to the patient or family members - Sometimes double administration results in sedation and there is significant **pharmacological constraints compared with single administration** - We found no difference from single administration in terms of side effects - Initial preconception - Subjective characteristics of the patient - Benefit in the patient's life |
| **No clear barrier or challenge identified/supplied (*n*=52)** | | - None - No - None - None - I have not encountered any difficulties - None - None - None - None - None - None - None - None - None - I don’t see any at the moment - None - N/A - None - None - None - [No obstacles or challenges that could be mentioned at the moment] - None - No specific one - No obstacles - None - No other - None - . - None - ....... - None - No obstacles to report - None - None - [Left blank] - No other - No obstacles in particular - No - N/A - - - None - None - None - None - None - . - None - None - . - I don't know, certainly patients manage their daily life much better - No difficulty - . |

Free-text responses were received in the participants’ local language and were translated into English where required.

Participant responses were grouped into common themes, shown in order from themes with the most to the least number of relevant responses (excluding the ‘Other’ and ‘No clear barrier or challenge identified/supplied’ themes, which are listed last).

Responses may have encompassed multiple themes; in such cases, they were included in more than one category (text shown in bold refers to the portion of a response that is relevant to the selected category).

N/A: not applicable; HCP: healthcare professional; NHS: National Health Service; TIS: two-injection start.

**Supplementary Figure 1.** Most common reason(s) selected by HCPs for initiating maintenance treatment with the AOM 400-TIS according to country

Data are ranked from the most to the least selected response options in Denmark. Participants could select all response options that applied.

^a^Other responses included: ‘side effect profile’ (*n*=1) [United Kingdom]; ‘simplification of the dosage scheme’ (*n*=1) [Italy]; ‘improved medication efficacy and tolerability’ (*n*=1) [Italy]; and ‘less degree of disease symptoms when taking pills daily’ (*n*=1) [Denmark].

AOM 400-TIS: aripiprazole once-monthly 400 mg two-injection start; HCP: healthcare professional.

**Supplementary Figure 2.** Main objective(s) selected by HCPs when choosing to prescribe the AOM 400-TIS initiation regimen according to country

Data are ranked from the most to least selected response options in Denmark. Participants could select all response options that applied.

^a^Other responses included: ‘improving tolerability’ (*n*=1) [Italy] and ‘stabilise serum values and hopes of less adverse reaction fluctuations’ (*n*=1) [Denmark]

AOM 400-TIS: aripiprazole once-monthly 400 mg two-injection start; HCP: healthcare professional.

**Supplementary Figure 3.** Reasons and/or barriers selected by HCPs that might lead them to not use the AOM 400-TIS initiation regimen according to country

Data are ranked from the most to least selected response options in Denmark. Participants could select all response options that applied.

^a^Other responses included: ‘based on how the question is phrased, I have no obstacles but other colleagues may not prescribe due to lack of specific training’ (*n*=1) [Italy]; ‘none’ (*n*=1) [Italy]; ‘I do not prescribe’ (*n*=1) [Italy]; ‘I administer by prescription’ (*n*=1) [Italy]; and ‘Nothing’ (*n*=1) [Sweden].

AOM 400-TIS: aripiprazole once-monthly 400 mg two-injection start; HCP: healthcare professional.

**Supplementary Figure 4.** Important patient and clinical factors that influence HCPs’ decision to prescribe the AOM 400-TIS initiation regimen according to country

Data are ranked from the most to least selected response options in Denmark. Participants could select all response options that applied.

AOM 400-TIS: aripiprazole once-monthly 400 mg two-injection start; HCP: healthcare professional.

**Supplementary Figure 5.** Top three most important patient and clinical factors that influence HCPs’ decision to prescribe the AOM 400-TIS initiation regimen

Participants were asked to select the response option that they considered the first, second, and third most important.

Data are ranked from the most to least selected response options in the most important category. The denominator was *n*=178 (all participants [*N*=229] minus those that did not prescribe as part of their job role [*n*=51]).

AOM 400-TIS: aripiprazole once-monthly 400 mg two-injection start; HCP: healthcare professional.

**Supplementary Figure 6.** HCP responses to other statements on recent experiences with the AOM 400-TIS initiation regimen (*N*=229)

AOM 400-TIS: aripiprazole once-monthly 400 mg two-injection start; HCP: healthcare professional.

**Reference**

1. Otsuka Pharmaceutical Netherlands B.V. Abilify Maintena^®^ (aripiprazole). Summary of product characteristics. October 2025. <https://www.ema.europa.eu/en/documents/product-information/abilify-maintena-epar-product-information_en.pdf>. Accessed January 20, 2026.
